# Assessing the fall risk with Stay Independent Questionnaire in people with stroke

**DOI:** 10.3389/fneur.2024.1476313

**Published:** 2025-01-03

**Authors:** Peiming Chen, T. W. Liu, Shamay S. M. Ng

**Affiliations:** ^1^Department of Rehabilitation Sciences, The Hong Kong Polytechnic University, Hung Hom, Hong Kong SAR, China; ^2^Research Centre for Chinese Medicine Innovation, The Hong Kong Polytechnic University, Hung Hom, Hong Kong SAR, China; ^3^School of Nursing and Health Studies, Hong Kong Metropolitan University, Kowloon, Hong Kong SAR, China

**Keywords:** stroke, lower limb, fall risk, balance, assessment

## Abstract

**Objectives:**

This study aimed to assess the psychometric properties of the Chinese version of the Stay Independent Questionnaire (C-SIQ) in evaluating individuals with stroke.

**Design:**

The study adopted a cross-sectional design.

**Setting:**

The research was conducted at a university-based neurorehabilitation center.

**Participants:**

The study included a total of 100 individuals with stroke and 49 healthy older adults.

**Methods:**

On Day 1, both individuals with stroke and healthy older adults underwent assessments using the C-SIQ. Additionally, individuals with stroke were evaluated using the Fugl–Meyer Assessment of Lower Extremity (FMA-LE), ankle dorsiflexion and plantarflexion strength, Berg Balance Scale (BBS), Timed-Up and Go Test (TUG), 10-meter walk test (10 mWT), Activities-specific Balance Confidence (ABC) Scale, Stroke Impact Scale (SIS), and Community Integration Measure (CIM). On Day 2 (7 days after Day 1), individuals with stroke were reassessed using the C-SIQ.

**Results:**

Individuals with stroke exhibited a higher C-SIQ score (6.22 ± 2.98) compared to healthy older adults (1.59 ± 2.01). The C-SIQ demonstrated good test–retest reliability (intraclass correlation coefficient = 0.847) and internal consistency (Cronbach’s alpha = 0.709). The Minimal Detectable Change in C-SIQ score was calculated as 3.05. Exploratory factor analysis revealed four factors with eigenvalues ≥1.0, explaining 57.17% of the total variance. The C-SIQ score exhibited significant correlations (ranging from −0.553 to 0.362) with completion times of the TUG and 10 mWT, FMA-LE, BBS, ABC, SIS, CIM score, paretic ankle dorsiflexion strength, and 6 mWT distance. A cut-off score of 2.5 was identified as the optimal threshold for discriminating fall risk between individuals with stroke and healthy controls.

**Conclusion:**

The C-SIQ emerges as a reliable and valid tool for evaluating fall risk in individuals with stroke, showcasing strong correlations with key measures such as TUG times, 10 mWT, FMA-LE, BBS, ABC, SIS, CIM score, paretic ankle dorsiflexion strength, and 6 mWT distance. The C-SIQ demonstrated good test–retest reliability and internal consistency. Exploratory factor analysis revealed that this is a four factors assessment tool. The identified cut-off score of 2.5 effectively distinguishes fall risk between individuals with stroke and healthy controls.

## 1 Introduction

Falls are among the most common incidents among people with stroke due to multiple factors (e.g., lower limb motor function, cognitive function) caused by brain lesions (1). Previous epidemiological studies (2) have indicated that the incident rate of falls ranges from 33 to 48% in the first year following stroke. The risk of fracture in people with stroke is 1.4–7 times higher than the general population (3, 4). The fracture could lead to decreased physical activity (5) and impede the restoration of independent daily living (6). According to a report by the World Health Organization, falls are associated with 684,000 deaths per year, which indicated the second leading causes of unintentional injury death (7). To reduce the incidence of falls in people with stroke, an effective assessment tool that can accurately evaluate fall risk should be identified.

According to the previous systematic review (8), fall risks are assessed via various aspects clinically, such as medical history questions, functional performance tests and comprehensive screening tools. Medical history question was one of the typical assessment components for screening the fall risk, which generally included the age, fall history, fear of fall level, use of ambulatory assistive device and use of medication. However, the pure medical history may only reflect the past situation of fall risk and not able to provide sufficient information of the current fall-related conditions. Some functional performance tests, such as Berg Balance Scale (BBS) (9) and Timed Up and Go (TUG) (10), can directly measure the potentially modifiable balance ability and functional mobility in everyday activities such as walking up and down stairs and mobility as well as the speed, and can indirectly reflect fall risk. However, the functional performance tests cannot directly assess the cognitive psychological factors, such as the executive function and memory, which showed strong correlation with fall risk (11). Up to now, there is no identified best single method (12), with current recommendations based on the combination of multiple methods (8, 13). If fall risk is suspected to be high, instead of using a single assessment tool, using several assessment tools that have distinctive characteristics to make multiple assessments of fall risk would be necessary to increase the overall predictive accuracy. Some comprehensive screening can achieve this objective to overcome the disadvantage of the previous physical examination. In the Johns Hopkins Fall Risk Assessment Tool (14), Hendrich II Fall Risk Model (15) and St. Thomas’s Risk Assessment Tool in Falling elderly inpatients (16), fall risk is assessed by medical staff (e.g., nurses) making decisions on fall risk, involving items such as conscious state, urinary function, patient care equipment and drug-taking. The process required specific expertise or specialized clinic equipment. While frequently applied within the clinical environments, it is not commonly applied in quotidian contexts. Therefore, to identify an easy-to-administrate assessment tool to evaluate the fall risk among people with stroke compressively is warranted.

The Stay Independent Questionnaire (SIQ) was developed from the Fall Risk Questionnaire (FRQ) (17), which is a multifactorial evaluation tool to assess risk factors for falls, including age, history of falls, bowel control, medication, patient care, mobility, and cognition, in community-dwelling older adults, which allows for more specialized health care for those at increased fall risk. Rubenstein et al. (18) modified the 13-item FRQ into a 12-item SIQ to evaluate fall risk and identified a cutoff score of ≥4 to distinguish between high and low fall risk in older adults. The assessment with SIQ did not require professional training or equipment. Due to its advantage, it was integrated as “Stay Independent Brochure” into the U.S. Centers for Disease Control’s Stopping Elderly Accidents, Death & Injuries program to increase older adults’ awareness of their personal fall risk. SIQ has shown excellent test–retest and inter-rater reliability [Intraclass Correlation Coefficient (ICC) = 0.89–0.95] in Thai’s elderly people (19). However, no study investigates its psychometric property to assess the fall risk in people with stroke.

Although the SIQ has been determined to be an effective assessment tool for evaluating fall risk, to the best of our knowledge, no study has translated the SIQ into Cantonese or culturally adapted it for the Chinese population and explore its psychometric property in people with stroke. In this study, the aim is to: (1) translate and culturally adapt the SIQ to assess fall risk in people with stroke in Hong Kong; (2) investigate the test–retest reliability and internal consistency of the Chinese version of the SIQ (C-SIQ); (3) identify the minimal detectable change (MDC) in the C-SIQ score in stroke; (4) investigate the factor structure of C-SIQ in people with stroke; (5) investigate the correlation between C-SIQ scores and other stroke-specific impairments; and (6) determine the cutoff score of the C-SIQ that differentiates the performance of people with stroke from that of healthy older adults.

## 2 Methods

### 2.1 Translation and cultural adaption

The original SIQ was translated to C-SIQ following the guideline of the forward-backward translation. At first, the English version of the SIQ was forward translated to Chinese by 2 independent native Cantonese speakers (F1: research assistant with rehabilitation background and F2: professional translator in the language center without rehabilitation background) with rehabilitation background. Two Chinese drafts were drafted by the 2 translators (F1 and F2). A consensus forward C-SIQ was then generated by the translator after resolving the discrepancies. The consensus Chinese version was then translated back to English by another 2 English bilingual translators (B1: physiotherapist and B2: professional translator in the language center without rehabilitation background). These two independent bilingual translators then address and resolve any linguistic discrepancies of the backward translated SIQ during the translation process. The expert panel with 6 members with rich research experience (registered nurse, mental health nurse, rehabilitation therapist, 2 professional research assistant) compared the backward translated SIQ with the original questionnaire to identify any discrepancies or areas requiring clarification. After checking the grammatical errors, typos and the formatting issue of the consensus backward translated version of the SIQ, the final version of C-SIQ was ready for data collection.

### 2.2 Sample size calculation

There was no previous study investigate the reliability of SIQ in people with stroke. We found an excellent test–retest reliability of SIQ when assessing healthy elderly people (19). Therefore, a conservative test–retest reliability (ICC = 0.90) was assumed in assessing the fall risk in people with stroke. In order to fulfill the requirement of 80% power and a significant level of 0.05, the minimum sample size was estimated to be 62. It was estimated by the online sample size calculation for reliability (20).

No previous study has investigated the correlation between SIQ and stroke-specific outcome measures in people with stroke. We hypothesize a weak correlation (*r* = 0.25) exists between SIQ and the stroke-specific outcome measures, a minimum sample size of 95 subjects was required to achieve 80% of power and a significant level of 0.05. It was conducted with the software G∗Power 3.1.9.7 (Franz Faul, University of Kiel, Kiel, Germany).

In order to make a more robust conclusion, we increased the sample size to 100 to assess the correlation between SIQ and the stroke-specific outcome measures.

### 2.3 Design

This cross-sectional study was conducted in the neurorehabilitation laboratory at The Hong Kong Polytechnic University. All participants received clear explanation of the objective and procedure of the study. Written informed consent was obtained from each participant prior to the commencement of data collection. This study was approved by the ethics committee of the local institution and conducted in accordance with the Declaration of Helsinki (21).

### 2.4 Participants

All participants were recruited from local self-help groups within the community through advertisement flyers. People with stroke were included if they (1) were aged over 50 years, (2) had received a diagnosis of a first stroke through magnetic resonance imaging or computed tomography more than 1 year prior, (3) had an Abbreviated Mental Test score of at least 7 (22), (4) had a stable medical condition, (5) had no severe deficits in verbal communication, (6) were able to provide informed consent, and (7) were not involved in other clinical or medicinal trials. Participants were excluded if they had any other neurological diseases or comorbidities (e.g., Parkinson’s disease, uncontrolled diabetes, and cardiovascular or musculoskeletal conditions) other than stroke that could hinder proper assessment in this study.

Healthy older adults were included using the same inclusion and exclusion criteria as for people with stroke, except that healthy older adults were required to have no history of stroke and no impairments that could hinder proper assessment in this study.

### 2.5 Procedure

**Figure 1:**
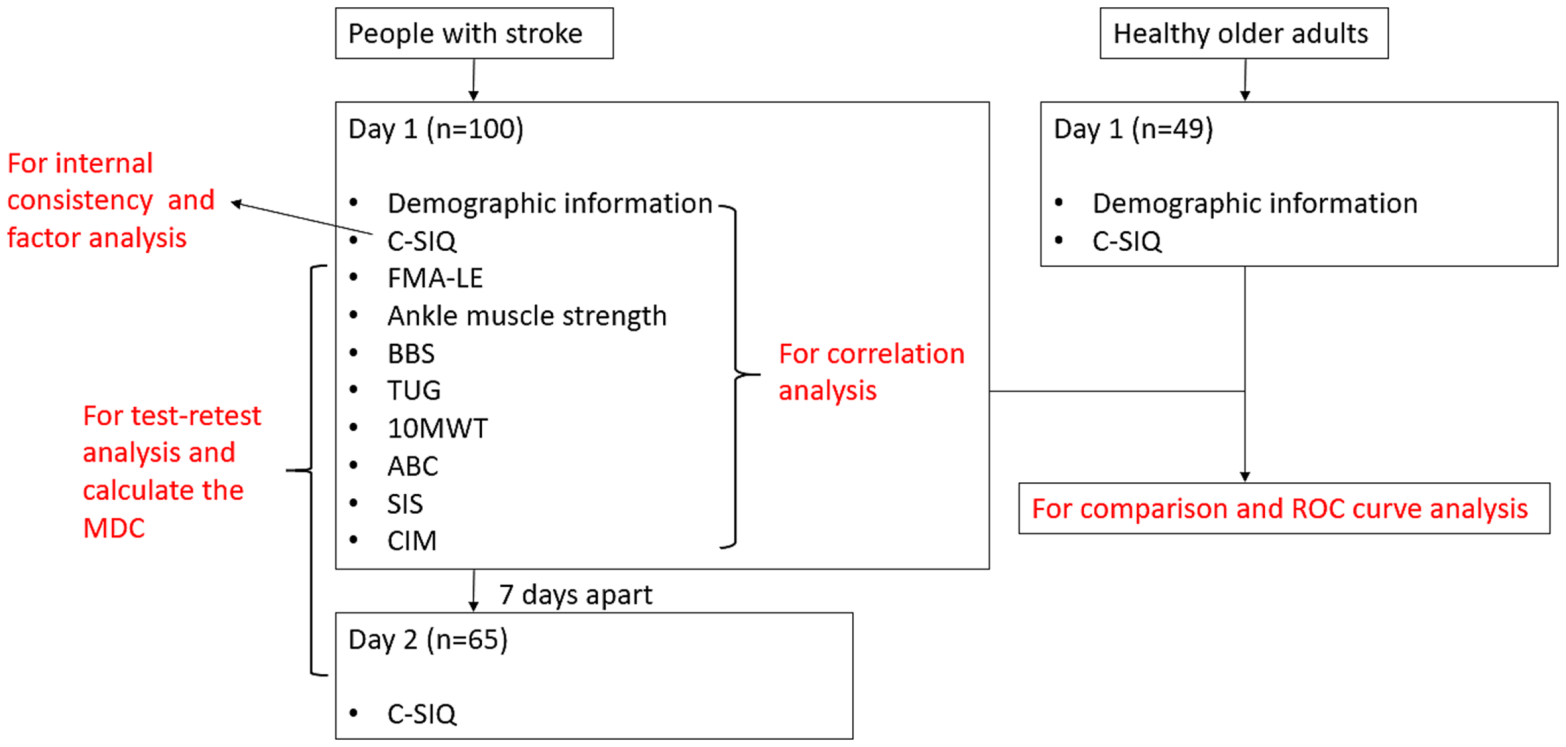
Flowchart of the study.

The testing procedure is illustrated in Figure 1. Five raters were trained, provided with clear instructions, and approved by the principal investigator prior to the commencement of the study. Thus, the rater will be independent in this study. The raters were blind to the study hypothesis. Each participant was assessed by the same rater on Day 1 and Day 2. For the people with stroke, assessments were conducted on both Day 1 and Day 2 (7 days after Day 1). On Day 1, the C-SIQ data and the demographic information were collected. According to the International Classification of Disability, Functioning and Health, the Fugl–Meyer Assessment of Lower Extremity (FMA-LE), muscle strength of ankle dorsiflexion and plantarflexion was used to assess the level of body function and body structure. The BBS, timed up and go test (TUG), 10-meter walk test (10 mWT), Activities-specific Balance Confidence (ABC) Scale was used to assess the activity level. The Stroke Impact Scale (SIS), and Community Integration Measure (CIM) were used to assess the participation level. The sequence of the assessments was randomized through a lucky draw to control for any learning effect among participants. In order to make the subjects in this study stable, a 3-min rest interval was provided between each motor task trial to eliminate the fatigue effect. For the healthy older adults, the C-SIQ was administered only on Day 1.

### 2.6 Outcome measures

#### 2.6.1 Chinese version of stay independent questionnaire (C-SIQ)

The C-SIQ is used to assess the risk of falls. This questionnaire evaluates the presence of various risk factors for falls, including the history of falls in the past year, use of a walking aid, gait and balance status, fear of falling, muscle weakness, medication use, and depression symptoms. Participants are required to respond with ‘yes’ or ‘no’ to each question. The C-SIQ is an ordinal scale comprising 12 items (items 1 and 2 are scored from 0 to 2 and items 3 to 12 are scored from 0 to 1). The total score ranges from 0 to 14. A total score of less than 4, between 4 and 8, and more than 8 indicates minimal or no risk of falls, a moderate to high risk of falls, and a high to severe risk of falls, respectively. The original SIQ exhibited an agreement with the clinical exam (kappa = 0.305–0.832, *p* < 0.05) in older adults (18).

#### 2.6.2 Fugl–Meyer assessment of lower extremity (FMA-LE)

The FMA-LE is used to assess lower limb motor control, including reflexes, coordination, and synergetic and isolated movements, in people with stroke (23). It is an ordinal scale comprising 17 items. Each item is scored from 0 to 2, with the total score ranging from 0 to 34. A higher FMA-LE score indicates better motor control of the lower limb. The FMA-LE was determined to have excellent reliability [intraclass correlation coefficient (ICC = 0.96)] in assessing patients with stroke (23).

#### 2.6.3 Muscle strength

A handheld dynamometer (model 01160; Lafayette Instrument Company, Lafayette, IN, USA) was used to assess the muscle strength of ankle dorsiflexion and plantarflexion (24). The participants assumed a supine lying position with 0° hip and knee flexion and their ankle in a neutral position. The dynamometer was placed on the mid-shaft area of the first to fifth metatarsal bones anteriorly or posteriorly to evaluate the strength of ankle dorsiflexion and plantarflexion, respectively. Make tests were performed on both the ankle dorsiflexor and plantarflexor muscles. During the test, a standard phrase of encouragement, ‘Push against my resistance as hard as you can’, was used. The participants were required to perform maximal voluntary isometric contractions for 3 s during each trial. To prevent muscle fatigue, a minimum rest period of 1 min was provided. The peak value of each trial was recorded, and the mean value of three trials was used for data analysis. The handheld dynamometer exhibited excellent test–retest reliability (ICC = 0.82–0.95) in assessing lower limb muscle strength in people with chronic stroke (25).

#### 2.6.4 Berg balance scale (BBS)

The BBS is used to assess functional balance (26). It is an ordinal scale comprising 14 functional balance tasks. Each task is scored from 0 to 4, with the total score ranging from 0 to 56. A higher BBS score indicates better functional balance. The BBS exhibited excellent inter-rater and intra-rater reliability (ICC = 0.97–0.98) in assessing functional balance in people with acute stroke (26).

#### 2.6.5 Timed-up and go test (TUG)

The TUG is used to assess functional mobility (27). During the test, the participants were required to stand up from a chair, walk 3 m, turn 180°, then walk back to the chair, and sit down. The completion time was recorded using a stop watch during each trial. Three trials of the TUG were performed for each participant. A shorter TUG completion time indicates better functional mobility. The TUG exhibited excellent test–retest reliability (ICC = 0.95–0.97) in people with stroke (27).

#### 2.6.6 10 meter walk test (10 mWT)

The 10 mWT is used to assess walking speed (28). The participants were asked to walk a distance of 10 m from a standing position at the comfortable and fast speed condition, respectively. The completion time was recorded using a stop watch during each condition. The average completion time was used for analysis. A shorter completion time indicated a faster walking speed. The 10 mWT demonstrated excellent test–retest reliability (ICC = 0.94–0.97) in people with stroke (29).

#### 2.6.7 6 minute walk test (6 mWT)

The 6 mWT is used to assess aerobic capacity and endurance (30). The 6 MWT was conducted in accordance to guidelines provided in the American Thoracic Society Statement (30). The participants were instructed to walk back and forth along a 30-m path as quickly as possible for 6 min. Standardized encouragements were provided at 1, 3, and 5 min during the walk: ‘You are doing a good job’ (minute 1), ‘You are halfway done’ (minute 3), and ‘You have 1 min to go’ (minute 5). The participants were allowed to stop and rest if required. The total distance covered in the 6 min was recorded. The 6 mWT demonstrated good intra-rater (ICC = 0.74) and inter-rater (ICC = 0.78) reliability in people with stroke (31).

#### 2.6.8 Activities-specific balance confidence scale (ABC)

The ABC Scale is used to assess balance confidence while performing daily functional activities (32). It is an ordinal scale comprising 16 items. Each item is scored from 0 (no confidence) to 100 (completely confident). A higher ABC score indicates a higher level of balance confidence. The mean score of 16 items was used for data analysis. The ABC Scale exhibited excellent reliability in people with stroke (33).

#### 2.6.9 Stroke impact scale (SIS)

The SIS is used to assess the health-related quality of life (34). This scale covers nine domains: strength (four items), hand function (five items), activity of daily living (ten items), mobility (nine items), communication (seven items), emotion (nine items), memory and thinking (seven items), and participation (eight items). Each item is scored from 1 to 5. Scores for each domain are converted to a score out of 100 using the following formula: [(mean domain score − 1)/(5–1)] × 100. A higher SIS score indicates a better quality of life. The SIS demonstrated good test–retest reliability (ICC = 0.7–0.92) in assessing people with stroke, except for the emotion domain (34).

#### 2.6.10 Community integration measure (CIM)

The CIM is used to assess the community integration level (35). It is an ordinal scale comprising 10 items. Each item is scored from 1 (always disagree) to 5 (always agree), with the total score ranging from 10 to 50. A higher CIM score indicates a better community integration level. The CIM showed good reliability (ICC = 0.84) in assessing people with stroke in a previous study (35).

### 2.7 Statistical analysis

Data analysis was conducted using SPSS version 26.0 (IBM Corporation, Armonk, New York, USA). Descriptive analysis was performed to summarize the demographic information of the participants. The Kolmogorov–Smirnov test was employed to examine the normality of data. The independent *t*-test, Mann–Whitney *U* test, and chi-square test were used to compare parametric and nonparametric data between people with stroke and healthy older adults as appropriate. The floor effect is the percentage of the sample scoring the minimum possible scores. The ceiling effect represents the opposite extreme. More than 15%, 10–15%, 5–10% and less than 5% were classified as significant, moderate, minor and negligible floor/ceiling effect (36).

The ICC value was calculated to assess test–retest reliability in people with stroke. As the subjects were assessed by a fixed rater, who was randomly selected from our research team, in Day 1 and Day 2, we used ICC 2, 1 (two-way random effects, absolute agreement, and single rater) to quantify the degree of test–retest reliability (37). An ICC value of <0.5, 0.5–0.75, 0.75–0.9, and > 0.9 indicates poor, moderate, good, and excellent reliability, respectively (37).

The MDC (95% confidence interval [CI]) was calculated to estimate the minimum change that would reflect a real change instead of a random error. The MDC was calculated as follows:


$$MDC\;\left(95\%CI\right)=1.96\times \sqrt{2}\times SD\times \sqrt{1-r}$$

where *SD* is the standard deviation of the C-SIQ score on Day 1 and Day 2, and *r* is the test–retest reliability of the C-SIQ score.

The Cronbach’s *α* coefficient was sued to assess the internal consistency of total score and each item score. The coefficient value of ≥0.9, 0.8–0.9, 0.7–0.8, 0.6–0.7, and ≤ 0.6 is considered as excellent, good, acceptable, questionable and poor (38).

The exploratory factor analysis (EFA) was used to determine the structural validity of C-SIQ in people with stroke. The Kaiser-Meyer-Olkin (KMO) coefficient and Bartlett’s test was used to evaluate the sphericity. A KMO value of 0.5 or higher and a significant Bartlett’s test of sphericity (*p* < 0.05) was considered suitable for conducting the EFA (39). Factors with eigenvalues greater than 1.0 were generally retained. The items with factor loading exceed 0.4 indicated a meaningful contribution to a specific factor (39).

Pearson’s r and Spearman’s *ρ* were used to determine the correlation between the C-SIQ score and other stroke-specific outcome measures for parametric and non-parametric data, respectively. A correlation value of <0.25, 0.25–0.49, 0.5–0.75, and > 0.75 indicates weak, moderate, good, and excellent correlation, respectively (40).

The receiver operating characteristic (ROC) curve was generated to determine the cutoff of the C-SIQ score to distinguish the performance of the people with stroke from that of healthy older adults. The optimal cutoff score was determined using Youden’s index as follows:


$${\text{Youden}}^{\prime }s\;\text{Index}=\text{sensitivity}+\text{specificity}-1.$$

The area under the curve (AUC) was calculated to quantify the discriminative accuracy. An AUC value of ≥0.9, 0.8 to <0.9, 0.7 to <0.8, 0.5 to <0.7, and ≤ 0.5 indicates outstanding, excellent, acceptable, poor, and no discriminative accuracy, respectively (41). The level of significance level was set at *α* = 0.05.

## 3 Results

### 3.1 Characteristics of the participants

The demographic data of the people with stroke and healthy older adults are listed in Table 1. We included 100 people with stroke (58 men and 42 women) and 49 healthy older adults (14 men and 35 women) in this study. No missing value should be handled. A significant difference (*p* < 0.001) in sex distribution was found between the people with stroke and healthy older adults. The mean ages of the people with stroke and healthy older adults were 63.71 ± 6.33 and 61.61 ± 7.63 years, respectively. The poststroke duration of the people with stroke was 81.14 ± 53.30 months.

**Table 1:** Demographics of stroke survivors and healthy older adults.

Descriptor	Stroke (*n* = 100)	Healthy (*n* = 49)	*p*
Age (y)	63.71 ± 6.33	61.61 ± 7.63	0.078
Sex (M/F)	58 / 42	14 / 35	<0.001*
Height (m)	1.62 ± 0.12	1.62 ± 0.09	0.880
Weight (kg)	64.42 ± 9.72	59.13 ± 11.47	0.004*
Body mass index (kg/m^2^)	25.24 ± 11.27	22.46 ± 3.15	0.093
Post-stroke duration (month)	81.14 ± 53.30	/	/
Paretic side (L/R)	46 / 54	/	/
Stroke nature (I/H)	68 / 32	/	/

Values are mean ± SD or as otherwise noted. F, female; M, male; L, left; R, right; I, ischemic; H, hemorrhagic.

### 3.2 Performance of the C-SIQ score and other outcomes in people with stroke and healthy older adults

The performance of the C-SIQ score in all the participants is presented in Table 2. People with stroke had a significantly higher C-SIQ score than did healthy older adults (6.22 ± 2.98 vs. 1.59 ± 2.01, *p* < 0.001). The C-SIQ score did not significantly differ between Day 1 (6.22 ± 2.98) and Day 2 (6.03 ± 2.92) in people with stroke (*p* = 0.940). The performance of the other outcomes is presented in Table 3.

**Table 2:** Mean score of Chinese version of stay independent questionnaire (C-SIQ).

	Stroke		Healthy	*p*	*p*
	Day 1 (*n* = 100)	Day 2 (*n* = 64)	Day 1 (*n* = 49)	(Day 1 Stroke vs Day 1 Healthy)	(Day 1 Stroke vs Day 2 Stroke)
C-SIQ	6.22 ± 2.98	6.03 ± 2.92	1.59 ± 2.01	<0.001	0.940

Values are mean ± SD.

**Table 3:** Mean Score of other outcome measures in people with stroke.

	Value (*n* = 100)
FMA-LE	26.05 ± 4.42
Ankle dorsiflexion strength, kg	10.90 ± 6.15
Ankle plantarflexion strength, kg	8.53 ± 5.20
BBS	49.87 ± 6.90
TUG, s	17.72 ± 14.28
10 MWT-comfortable, s	24.38 ± 19.65
10 MWT-fast, s	19.92 ± 14.57
6 MWT, m	222.55 ± 92.11
ABC	64.07 ± 23.43
SIS	
Strength	37.18 ± 24.09
Hand function	80.04 ± 19.31
Activity of daily living	71.00 ± 18.35
Mobility	87.75 ± 15.26
Communication	80.60 ± 18.34
Emotion	82.31 ± 16.67
Memory and thinking	40.10 ± 35.29
Participation	70.47 ± 20.03
CIM	40.42 ± 7.07

Values are mean ± SD. ABC, Activities-specific Balance Confidence Scale; BBS, Berg Balance Scale; CIM, Community Integration Measure; FMA-LE, Fugl-Meyer Assessment of Lower Extremity; SIS, Stroke Impact Scale; TUG, Time-Up and Go Test; 10 MWT, 10 Meter Walk Test.

The C-SIQ score had the negligible ceiling effects (3%) and floor effect (5%) in people with stroke.

### 3.3 Test–retest reliability and MDC of the C-SIQ in people with stroke

The test–retest reliability and MDC are depicted in Table 4. The C-SIQ exhibited good test–retest reliability (ICC = 0.847) in evaluating people with stroke in this study. The MDC of the C-SIQ score in people with stroke was 3.05.

**Table 4:** Test–retest reliability and minimal detectable change (MDC) of C-SIQ.

	ICC	*p*	MDC
C-SIQ	0.847 (0.761–0.904)	<0.001	3.05 s

Values are ICC_2,1_ (95% CI). CI, confidence interval.

### 3.4 Internal consistency of C-SIQ in people with stroke

The C-SIQ score showed acceptable internal consistency (Cronbach’ *α* = 0.709). The internal consistency of each item was ranged from 0.671 to 0.707.

### 3.5 Exploratory factor analysis (EFA) of C-SIQ in people with stroke

A KMO test for sampling adequacy (KMO = 0.668) and a significance in Bartlett’s test of sphericity (*p* < 0.001) were conducted to verify the suitability of the data for EFA. These results indicated the data was suitable for the EFA. Initially, the EFA extracted 4 factors with eigenvalues ≥1.0, which accounted for 57.17% of the total variance. Factor loadings ranged from 0.454 to 0.929 after rotating the 4 factors. The C-SIQ consists of 4 factors: (factor 1, items 2–5 and 7; factor loadings ranged from 0.454 to 0.771; factor 2, items 6, 8–10; factor loadings ranged from 0.478 to 0.694; factor 3, items 11–12; factor loadings ranged from 0.692 to 0.833; factor 4, items 1; factor loadings 0.929) (see Table 5).

**Table 5:** Exploratory factor analysis for C-SIQ.

Items	Mean	SD	Factors (Varimax rotated)				Communalities
			1	2	3	4	
Item 1	0.4	0.80				0.929	0.122
Item 2	1.58	0.82	0.690				0.578
Item 3	0.72	0.45	0.771				0.770
Item 4	0.39	0.49	0.591				0.876
Item 5	0.69	0.47	0.747				0.610
Item 6	0.41	0.49		0.694			0.744
Item 7	0.42	0.50	0.454				0.784
Item 8	0.45	0.50		0.478			0.556
Item 9	0.64	0.48		0.636			0.390
Item 10	0.18	0.39		0.653			0.795
Item 11	0.13	0.34			0.833		0.674
Item 12	0.21	0.41			0.692		0.756
Initial Eigenvalues			3.046	1.539	1.164	1.112	
Variance Explained %			25.383	12.827	9.697	9.264	57.17%

Principal component analysis: Factor loadings that <0.4 were not shown.

### 3.6 Correlation between the C-SIQ score and other outcome measures

The correlation between the C-SIQ score and other outcome measures is shown in Table 6. The C-SIQ score exhibited a significant positive correlation (*r* = 0.314–0.362) with the completion times of the TUG and 10 mWT. The C-SIQ score displayed a significant negative correlation (*r* = −0.145 to −0.553) with the FMA-LE, BBS, ABC, SIS, CIM score, paretic ankle dorsiflexion strength, and 6 mWT distance.

**Table 6:** Correlations of C-SIQ score with other stroke-specific impairment outcome measures.

	*r*	*p*
FMA-LE	−0.379*	<0.001
Paretic side ankle dorsiflexion strength	−0.214*	0.032
Paretic side ankle plantarflexion strength	−0.145	0.149
BBS	−0.320*	0.001
TUG	0.362*	<0.001
10 MWT-comfortable	0.347*	<0.001
10 MWT-fast	0.314*	0.002
6 MWT	−0.342*	<0.001
ABC	−0.553*	<0.001
SIS	−0.522*	<0.001
Strength	−0.236*	0.018
Hand function	−0.217*	0.030
Activity of daily living	−0.436*	<0.001
Mobility	−0.293*	0.003
Communication	−0.409*	<0.001
Emotion	−0.521*	<0.001
Memory and thinking	−0.300*	0.002
Participation	−0.460*	<0.001
CIM	−0.300*	0.002

Correlation are spearman’s rho*indicated *p* < 0.05. ABC, Activities-specific Balance Confidence Scale; BBS, Berg Balance Scale; CIM, Community Integration Measure; FMA-LE, Fugl-Meyer Assessment of Lower Extremity; SIS, Stroke Impact Scale; TUG, Time-Up and Go Test; 10 MWT, 10 Meter Walk Test.

### 3.7 ROC curve of the C-SIQ

**Figure 2:**
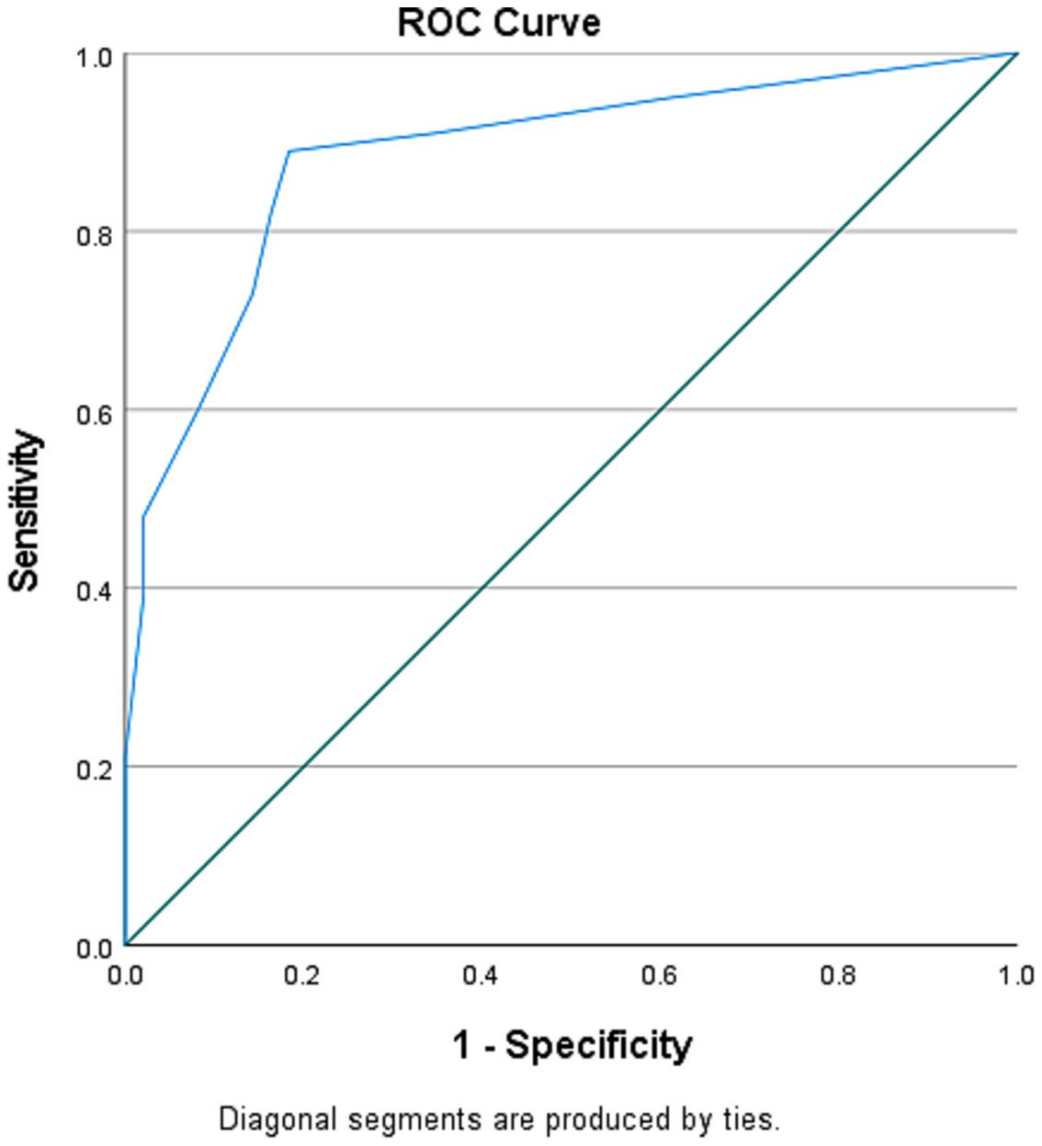
Receiver operating characteristic (ROC) curve for C-SIQ score for distinguishing the fall risk of people with stroke from healthy older adults [area under the curve (AUC) = 0.885; sensitivity = 89.0%; specificity = 81.6%; *p* < 0.001].

The C-SIQ cutoff score of 2.5 (AUC = 88.5%, sensitivity = 89.0%, specificity = 81.6%, *p* < 0.001) was identified as the best to distinguish the performance of people with stroke from that of healthy older adults (Figure 2).

## 4 Discussion

### 4.1 Summary of the finding

To the best of our knowledge, this is the first study to investigate the psychometric property of the C-SIQ in people with stroke. The C-SIQ exhibited good test–retest reliability in assessing people with stroke. The C-SIQ demonstrated good test–retest reliability and internal consistency. Exploratory factor analysis revealed that this is a four factors assessment tool. Moreover, the C-SIQ score was significantly correlated with the FMA-LE, BBS, ABC, SIS, and CIM scores, paretic ankle dorsiflexion strength, TUG and 10 mWT completion time, and the 6 mWT total distance. An optimal cutoff score of 2.5 was identified to distinguish the performance of the healthy older adults in our sample from that of people with stroke.

### 4.2 Performance of the C-SIQ

This is the first study to investigate the performance of the C-SIQ in people with stroke. The mean FRQ score (6.0) of people with osteoporosis in a previous study (42) is comparable to the mean C-SIQ score (6.05 to 6.22) of people with stroke in our study. Stroke often results in walking disability due to muscle weakness, spasticity (43), and sensory impairment (44). In osteoporosis, the substantial loss of bone mass and deterioration of bone tissue microarchitecture can reduce bone quality and strength, thereby increasing fracture risk (45). Both stroke and osteoporosis can lead to a diminished walking capacity in the elderly, increasing the risk of falls in daily life.

In addition, we found that the mean C-SIQ score was significantly higher in people with stroke than in healthy older adults (mean difference = 3.48). The mean difference in the C-SIQ score between the two groups exceeded the MDC calculated in our study. This notable difference likely arises from the distinct impact of stroke on lower limb motor function. Stroke can induce muscle weakness, coordination issues, and altered motor control, all of which can markedly affect performance on the C-SIQ test. Thus, the difference noted in the performance of the C-SIQ can be attributed to the real difference in lower limb motor function caused by stroke rather than measurement error (46). This discrepancy underscores the clinical utility of the C-SIQ as a sensitive tool for assessing lower limb motor function in people with stroke and emphasizes the need to consider the physiological changes in this population when interpreting test outcomes.

This is the first study to investigate the ceiling and floor effect of C-SIQ in people with stroke, our finding indicated that the C-SIQ showed negligible ceiling and floor effect in assessing the fall risk in people with stroke. It can therefore effectively capture fall risk across a wide spectrum of individuals post-stroke, without being limited by an inability to differentiate between those at the highest or lowest fall risk. This enhances the accuracy of fall risk assessment and ensures that individuals with varying levels of risk are appropriately identified and targeted for interventions.

### 4.3 Reliability

This is the first study to investigate the reliability of the C-SIQ in people with stroke. The findings revealed a good test–retest reliability of the C-SIQ. This result aligns with that of a previous study (42), which indicated an excellent agreement of FRQ in people with osteoporosis (kappa = 1). Several factors may explain the good test–retest reliability observed in this study. First, all participants in our study had received a diagnosis of chronic stroke, indicating that their motor function had achieved a stable level. Second, the provision of clear instructions to the participants regarding the study protocol and the adequate pretraining of raters substantially contributed to the excellent test–retest reliability. Third, the retest of the C-SIQ was performed 7 days after the initial test. A previous study (47) suggested that this 7-day test–retest interval between tests could effectively minimize the practice and cultivation effects on participants’ performance.

### 4.4 Internal consistency of C-SIQ in people with stroke

The C-SIQ score showed acceptable internal consistency. According to the guideline, the internal consistency of our finding was comparable with Loonlawong’s study (19) (Cronbach’ *α* = 0.78) in Thai’s population, which can be classified as acceptable. The similar finding can be contributed to the similar structure and proper translation of the questionnaire. Thus, we can find a consistent response.

### 4.5 EFA of C-SIQ in people with stroke

This is the first study to find a conduct the EFA for C-SIQ. The Exploratory Factor Analysis (EFA) revealed that the Structure of Inquiry Questionnaire (SIQ) demonstrates a 4-factor arrangement among individuals with stroke, contrasting with the 6-factor configuration observed in elderly Thai individuals (19). This variance could be attributed to varying responses to the SIQ across different populations. Individuals with stroke likely exhibit distinct risk factors compared to the elderly participants. Furthermore, our study had a smaller sample size of 100 participants, in contrast to the sample size of 480 in Loonlawong’s study (19). A larger sample size could enhance the identification of factors within the SIQ.

### 4.6 Correlation between the C-SIQ score and other outcome measures

#### 4.6.1 FMA-LE

This study revealed a significant correlation between the C-SIQ and FMA-LE scores. A previous study identified motor impairment as a clinical predictor of fall risk. Impaired muscle control, movement, and mobility can affect movement control on the paretic side (48), thus directly increasing the risk of falls. Therefore, the C-SIQ score was significantly correlated with the FMA-LE score in people with stroke.

#### 4.6.2 Ankle muscle strength

This study indicated a significant correlation between the C-SIQ score and ankle dorsiflexion strength, whereas the correlation between the C-SIQ score and ankle plantarflexion strength was nearly significant. This finding suggests that both the muscle strength of ankle dorsiflexion and plantarflexion are associated with fall risk. However, ankle dorsiflexion strength may play a more pivotal role in fall risk than ankle plantarflexion strength. A randomized controlled trial (49) found that when a postural disturbance occurs, an individual can recover balance and prevent a fall using a fixed-support reaction strategy. This strategy relies on the generation of muscle torque from the ankle and hip joints to slow down or stop the center of mass movement without changing the location and size of the base of support (50). Therefore, sufficient ankle muscle strength can contribute to trunk stabilization and fall prevention (51).

#### 4.6.3 BBS

The findings revealed that the BBS score was significantly correlated with the C-SIQ score in this study. This result is consistent with those of two previous studies involving healthy older adults (19) and older adults with knee osteoporosis (42), which showed a moderate significant correlation between the SIQ and BBS scores. A previous systematic review (52) revealed that, as a gold standard for assessing balance dysfunction in people with stroke, the BBS was a strong predictor (*r* = 0.797) of fall risk during the inpatient period. In addition, a 13-month longitudinal study of 80 people with stroke found that the BBS score was a significant predictor (*β* = −0.096, incidence rate ratio = 0.908) of the number of falls (53). Given that the C-SIQ evaluates fall risk in people with stroke, the BBS score is expected to be significantly correlated with the C-SIQ score in this population.

#### 4.6.4 TUG, 10 mWT, and 6 mWT

The TUG and 10 mWT completion time and 6 mWT total distance were significantly correlated with the C-SIQ score in this study. This finding is consistent with a previous study (42), which found that the FRQ was significantly correlated with TUG completion time in people with osteoporosis. A meta-analysis (8) reported that performance-based tests, such as the TUG, were a strong predictor of falls. Given that the TUG, 10 mWT, and 6 mWT are performance-based tests used to assess different domains (functional mobility, walking speed, and aerobic capacity) of locomotor function, it is reasonable to observe a significant correlation between the C-SIQ score and performance in the TUG, 10 mWT and 6 mWT.

#### 4.6.5 ABC

The findings of this study revealed that the ABC score was significantly correlated with the C-SIQ score. This finding is reasonable given that an increased fall rate can reduce balance confidence. A previous study indicated that balance confidence represents the cognitive component of fall risk, where older adults subjectively estimate their ability to prevent a fall or maintain balance (54).

#### 4.6.6 SIS

The SIS score was significantly correlated with the C-SIQ score in this study. The SIS is used to assess health-related quality of life, and the C-SIQ is primarily used to evaluate fall risk. A previous study (55) indicated that the risk of falls was correlated with the quality of life in older adults. Older adults who are dependent on others for basic daily activities due to difficulties with gait and impaired physical mobility are more likely to experience falls than those who are less dependent on others for support (56).

#### 4.6.7 CIM

The CIM score was significantly correlated with the C-SIQ score in this study. A meta-analysis (1) identified disability in self-care as a risk factor (odds ratio = 2.30) for falls in community-dwelling stroke survivors. Individuals with a high risk of falls after stroke may experience difficulties in performing self-care daily activities, such as bathing, toileting, and shopping. Another study (57) found that a high risk of falls can lead to increased economic and social costs, which can make reintegration into the community challenging. All these factors may directly or indirectly impede the reintegration of people with stroke into community living.

### 4.7 Optimal cutoff score of the C-SIQ

Compared with a study investigating the C-SIQ in healthy older adults (cutoff score > 4) (18), this study identified a lower cutoff score of >2.5 to distinguish the performance of healthy older adults from that of people with stroke. Compared with healthy older adults with a high risk of falls, people with stroke had poorer motor function in this study, resulting in a higher risk of falls than that observed in the study conducted by Rubenstein (18). In addition, the high AUC in this study revealed that the C-SIQ correctly distinguished the fall risk of healthy older adults from that of people with stroke with 88.5% accuracy. The C-SIQ demonstrated high sensitivity and specificity in assessing fall risk in people with stroke, indicating its potential as an effective assessment tool to differentiate between people with stroke and healthy older adults.

### 4.8 Clinical implication

Stroke survivors often face challenges such as muscle weakness, impaired coordination, and altered motor control, which directly affect their performance on the C-SIQ. By recognizing and interpreting these differences accurately, clinicians can tailor rehabilitation interventions more effectively to address the unique motor impairments experienced by people with stroke.

### 4.9 Limitation

This study has several limitations that should be addressed. First, the participants in this study were recruited from local self-help groups. Most of these participants were socially active and could live independently. Thus, the findings of this study may not be generalizable to the broader stroke population. Future studies should include more patients with different levels of motor impairment, such as the people who are ambulatory, community dwelling, several years following stroke. To involve these population would enhance the generalizability of the findings and provide clinicians with a more accurate understanding of C-SIQ in assessing the fall risk in the common stroke population. Second, the sample size may not be sufficient to detect significant correlations with some outcomes. Future studies with larger sample sizes are warranted to increase statistical power. Third, a significant difference was observed in both gender distribution between the stroke and the healthy participants. Previous study (58) have shown that biological and physiological factors related to gender can impact aspects such as muscle strength, coordination, balance, and motor control in the lower limbs. This factor could potentially influence walking performance. Precaution should be taken before interpreting the results in light of these differences.

## 5 Conclusion

The C-SIQ is a convenient tool for assessing fall risk in people with stroke. Future studies should encompass a broader spectrum of patients exhibiting varying degrees of motor impairment within the stroke population across diverse settings, including ambulatory individuals residing in the community and those several years post-stroke.

## Data Availability

The raw data supporting the conclusions of this article will be made available by the authors, without undue reservation.

## References

[1] Xu T Clemson L O’Loughlin K Lannin NA Dean C Koh G. Risk factors for falls in community stroke survivors: a systematic review and meta-analysis. Arch Phys Med Rehabil. (2018) 99:563–573.e5. doi: 10.1016/j.apmr.2017.06.032, PMID: 28797618

[2] Minet LR Peterson E Von Koch L Ytterberg C. Occurrence and predictors of falls in people with stroke: six-year prospective study. Stroke 46 (2015) 2688-2690,C.U. Persson, P.-O. Hansson, and K.S. Sunnerhagen, clinical tests performed in acute stroke identify the risk of falling during the first year: postural stroke study in Gothenburg (POSTGOT). J Rehabil Med. (2011) 43:348–53.21267528

[3] Pouwels S Lalmohamed A Leufkens B de Boer A Cooper C van Staa T. Risk of hip/femur fracture after stroke: a population-based case-control study. Stroke. (2009) 40:3281–5. 19661475 10.1161/STROKEAHA.109.554055

[4] Kapral MK Fang J Alibhai SM Cram P Cheung AM Casaubon LK. Risk of fractures after stroke: results from the Ontario stroke registry. Neurology. (2017) 88:57–64. doi: 10.1212/WNL.0000000000003457 27881629 PMC5200858

[5] Mackintosh SF Hill K Dodd KJ Goldie P Culham E. Falls and injury prevention should be part of every stroke rehabilitation plan. Clin Rehabil. (2005) 19:441–51. doi: 10.1191/0269215505cr796oa, PMID: 15929514

[6] Schmid AA Rittman M. Consequences of poststroke falls: activity limitation, increased dependence, and the development of fear of falling. Am J Occup Ther. (2009) 63:310–6. doi: 10.5014/ajot.63.3.310, PMID: 19522139

[7] World Health Organization. Fall. Geneva, Switzerland: World Health Organization (2021).

[8] Lusardi MM Fritz S Middleton A Allison L Wingood M Phillips E. Determining risk of falls in community dwelling older adults: a systematic review and meta-analysis using posttest probability. J Geriatr Phys Ther. (2017) 40:1–36. doi: 10.1519/JPT.0000000000000099, PMID: 27537070 PMC5158094

[9] Berg T. Berg balance scale. Arch Phys Med Rehabil. (2009) 73:2–5.

[10] Schoppen T Boonstra A Groothoff JW de Vries J Göeken LN Eisma WH. The timed “up and go” test: reliability and validity in persons with unilateral lower limb amputation. Arch Phys Med Rehabil. (1999) 80:825–8. doi: 10.1016/S0003-9993(99)90234-4, PMID: 10414769

[11] Muir SW Gopaul K Montero Odasso MM. The role of cognitive impairment in fall risk among older adults: a systematic review and meta-analysis. Age Ageing. (2012) 41:299–308. doi: 10.1093/ageing/afs012, PMID: 22374645

[12] Gates S Smith LA Fisher JD Lamb SE. Systematic review of accuracy of screening instruments for predicting fall risk among independently living older adults. Database of Abstracts of Reviews of Effects. (2008) 45:1105–16. 19235113

[13] Park S-H. Tools for assessing fall risk in the elderly: a systematic review and meta-analysis. Aging Clin Exp Res. (2018) 30:1–16. doi: 10.1007/s40520-017-0749-0, PMID: 28374345

[14] Poe SS Cvach M Dawson PB Straus H Hill EE. The Johns Hopkins fall risk assessment tool: postimplementation evaluation. J Nurs Care Qual. (2007) 22:293–8. doi: 10.1097/01.NCQ.0000290408.74027.39, PMID: 17873724

[15] Zhang C Wu X Lin S Jia Z Cao J. Evaluation of reliability and validity of the Hendrich II fall risk model in a Chinese hospital population. PLoS One. (2015) 10:e0142395. doi: 10.1371/journal.pone.0142395, PMID: 26544961 PMC4636230

[16] Webster J Courtney M Marsh N Gale C Abbott B Mackenzie-Ross A. The STRATIFY tool and clinical judgment were poor predictors of falling in an acute hospital setting. J Clin Epidemiol. (2010) 63:109–13. doi: 10.1016/j.jclinepi.2009.02.003, PMID: 19398296

[17] Vivrette RL Rubenstein LZ Martin JL Josephson KR Kramer BJ. Development of a fall-risk self-assessment for community-dwelling seniors. J Aging Phys Act. (2011) 19:16–29. doi: 10.1123/japa.19.1.16, PMID: 21285473 PMC3383800

[18] Rubenstein LZ Vivrette R Harker JO Stevens JA Kramer BJ. Validating an evidence-based, self-rated fall risk questionnaire (FRQ) for older adults. J Saf Res. (2011) 42:493–9. doi: 10.1016/j.jsr.2011.08.006, PMID: 22152267

[19] Loonlawong S Limroongreungrat W Jiamjarasrangsi W. The stay independent brochure as a screening evaluation for fall risk in an elderly Thai population. Clin Interv Aging. (2019) 14:2155–62. doi: 10.2147/CIA.S233414, PMID: 31849459 PMC6913248

[20] Walter S Eliasziw M Donner A. Sample size and optimal designs for reliability studies. Stat Med. (1998) 17:101–10. doi: 10.1002/(SICI)1097-0258(19980115)17:1<101::AID-SIM727>3.0.CO;2-E, PMID: 9463853

[21] W.M. Association. World medical association declaration of Helsinki: ethical principles for medical research involving human subjects. JAMA. (2013) 310:2191–4. doi: 10.1001/jama.2013.281053 24141714

[22] Lam SC Wong YY Woo J. Reliability and validity of the abbreviated mental test (Hong Kong version) in residential care homes. J Am Geriatr Soc. (2010) 58:2255–7. doi: 10.1111/j.1532-5415.2010.03129.x, PMID: 21054326

[23] Duncan PW Propst M Nelson SG. Reliability of the Fugl-Meyer assessment of sensorimotor recovery following cerebrovascular accident. Phys Ther. (1983) 63:1606–10. doi: 10.1093/ptj/63.10.1606, PMID: 6622535

[24] Eng JJ Kim CM MacIntyre DL. Reliability of lower extremity strength measures in persons with chronic stroke. Arch Phys Med Rehabil. (2002) 83:322–8. doi: 10.1053/apmr.2002.29622, PMID: 11887111 PMC3489912

[25] Kluding P Gajewski B. Lower-extremity strength differences predict activity limitations in people with chronic stroke. Phys Ther. (2009) 89:73–81. doi: 10.2522/ptj.20070234, PMID: 18988665

[26] Berg K Wood-Dauphinee S Williams J. The balance scale: reliability assessment with elderly residents and patients with an acute stroke. Scand J Rehabil Med. (1995) 27:27–36. doi: 10.2340/1650197719952736, PMID: 7792547

[27] Ng SS Hui-Chan CW. The timed up & go test: its reliability and association with lower-limb impairments and locomotor capacities in people with chronic stroke. Arch Phys Med Rehabil. (2005) 86:1641–7. doi: 10.1016/j.apmr.2005.01.011, PMID: 16084820

[28] Vos-Vromans DC de Bie RA Erdmann PG van Meeteren NL. The responsiveness of the ten-meter walking test and other measures in patients with hemiparesis in the acute phase. Physiother Theory Pract. (2005) 21:173–80. doi: 10.1080/09593980500212920, PMID: 16389698

[29] Flansbjer U-B Holmbäck AM Downham D Patten C Lexell J. Reliability of gait performance tests in men and women with hemiparesis after stroke. J Rehabil Med. (2005) 37:75–82. PMID: 15788341 10.1080/16501970410017215

[30] Enright PL. The six-minute walk test. Respir Care. (2003) 48:783–5. PMID: 12890299

[31] Marc Kosak M Smith T. Comparison of the 2-, 6-, and 12-minute walk tests in patients with stroke. J Rehabil Res Dev. (2005) 42:103–7. doi: 10.1682/jrrd.2003.11.0171 15742254

[32] Mak MK Lau AL Law FS Cheung CC Wong IS. Validation of the Chinese translated activities-specific balance confidence scale. Arch Phys Med Rehabil. (2007) 88:496–503. doi: 10.1016/j.apmr.2007.01.018, PMID: 17398252

[33] Botner EM Miller WC Eng JJ. Measurement properties of the activities-specific balance confidence scale among individuals with stroke. Disabil Rehabil. (2005) 27:156–63. doi: 10.1080/09638280400008982, PMID: 15824045

[34] Vellone E Savini S Fida R Dickson VV Melkus GDE Carod-Artal FJ. Psychometric evaluation of the stroke impact scale 3.0. J Cardiovasc Nurs. (2015) 30:229–41. doi: 10.1097/JCN.0000000000000145, PMID: 24695074

[35] Liu T-W Ng SS Ng GY. Translation and initial validation of the Chinese (Cantonese) version of community integration measure for use in patients with chronic stroke. Biomed Res Int. (2014) 2014:1–7. doi: 10.1155/2014/623836, PMID: 24995317 PMC4065661

[36] Gulledge CM Smith DG Ziedas A Muh SJ Moutzouros V Makhni EC. Floor and ceiling effects, time to completion, and question burden of PROMIS CAT domains among shoulder and knee patients undergoing nonoperative and operative treatment. JBJS Open Access. (2019) 4:e0015. doi: 10.2106/JBJS.OA.19.00015, PMID: 32043052 PMC6959920

[37] Koo TK Li MY. A guideline of selecting and reporting intraclass correlation coefficients for reliability research. J Chiropr Med. (2016) 15:155–63. doi: 10.1016/j.jcm.2016.02.012, PMID: 27330520 PMC4913118

[38] George D. SPSS for windows step by step: A simple study guide and reference, 17.0 update, 10/e. Boston, United States: Pearson Education India (2011).

[39] Williams B Onsman A Brown T. Exploratory factor analysis: a five-step guide for novices. Austr J Paramed. (2010) 8:1–13. doi: 10.33151/ajp.8.3.93

[40] Portney LG Watkins MP. Foundations of clinical research: Applications to practice. NJ: Pearson/Prentice Hall Upper Saddle River (2009).

[41] Streiner DL Cairney J. What’s under the ROC? An introduction to receiver operating characteristics curves. Can J Psychiatry. (2007) 52:121–8. doi: 10.1177/070674370705200210, PMID: 17375868

[42] Kitcharanant N Vanitcharoenkul E Unnanuntana A. Validity and reliability of the self-rated fall risk questionnaire in older adults with osteoporosis. BMC Musculoskelet Disord. (2020) 21:1–9. doi: 10.1186/s12891-020-03788-z PMC7677850 33208120

[43] Xie T Leng Y Xu P Li L Song R. Mapping of spastic muscle activity after stroke: difference between passive stretch and active contraction. J Neuroeng Rehabil. (2024) 21:102. doi: 10.1186/s12984-024-01376-z, PMID: 38877589 PMC11177522

[44] Kwong PW Ng GY Chung RC Ng SS. Bilateral transcutaneous electrical nerve stimulation improves lower-limb motor function in subjects with chronic stroke: a randomized controlled trial. J Am Heart Assoc. (2018) 7:e007341. doi: 10.1161/JAHA.117.007341, PMID: 29437598 PMC5850185

[45] Moreira LDF Oliveira M Lirani-Galvão A Marin-Mio R Santos R Lazaretti-Castro M. Physical exercise and osteoporosis: effects of different types of exercises on bone and physical function of postmenopausal women. Arquivos Brasileiros Endocrinol Metabol. (2014) 58:514–22. doi: 10.1590/0004-2730000003374, PMID: 25166042

[46] Furlan L Sterr A. The applicability of standard error of measurement and minimal detectable change to motor learning research—a behavioral study. Front Hum Neurosci. (2018) 12:95. doi: 10.3389/fnhum.2018.00095, PMID: 29623034 PMC5875129

[47] Marx RG Menezes A Horovitz L Jones EC Warren RF. A comparison of two time intervals for test-retest reliability of health status instruments. J Clin Epidemiol. (2003) 56:730–5. doi: 10.1016/S0895-4356(03)00084-2, PMID: 12954464

[48] Langhorne P Coupar F Pollock A. Motor recovery after stroke: a systematic review. Lancet Neurol. (2009) 8:741–54. doi: 10.1016/S1474-4422(09)70150-4 19608100

[49] Junata M Cheng KC-C Man HS Lai CW-K Soo YO-Y Tong RK-Y. Kinect-based rapid movement training to improve balance recovery for stroke fall prevention: a randomized controlled trial. J Neuroeng Rehabil. (2021) 18:1–12. doi: 10.1186/s12984-020-00774-3, PMID: 34635141 PMC8503723

[50] Chen N Xiao X Hu H Chen Y Song R Li L. Identify the alteration of balance control and risk of falling in stroke survivors during obstacle crossing based on kinematic analysis. Front Neurol. (2019) 10:813. doi: 10.3389/fneur.2019.00813, PMID: 31417488 PMC6682676

[51] Maki BE McIlroy WE. Control of rapid limb movements for balance recovery: age-related changes and implications for fall prevention. Age Ageing. (2006) 35:ii12–ii18. doi: 10.1093/ageing/afl078, PMID: 16926197

[52] Maeda N Urabe Y Murakami M Itotani K Kato J. Discriminant analysis for predictor of falls in stroke patients by using the Berg balance scale. Singapore Med J. (2015) 56:280–3. doi: 10.11622/smedj.2015033, PMID: 25678051 PMC4447930

[53] Simpson LA Miller WC Eng JJ. Effect of stroke on fall rate, location and predictors: a prospective comparison of older adults with and without stroke. PLoS One. (2011) 6:e19431. doi: 10.1371/journal.pone.0019431, PMID: 21559367 PMC3084849

[54] Ekechukwu N Olaleye O Hamzat T. Clinical and psychosocial predictors of community reintegration of stroke survivors three months post in-hospital discharge. Ethiop J Health Sci. (2017) 27:27–34. doi: 10.4314/ejhs.v27i1.5, PMID: 28458488 PMC5390226

[55] Scaf-Klomp W Sanderman R Ormel J Kempen GI. Depression in older people after fall-related injuries: a prospective study. Age Ageing. (2003) 32:88–94. doi: 10.1093/ageing/32.1.88, PMID: 12540354

[56] Ruthazer R Lipsitz LA. Antidepressants and falls among elderly people in long-term care. Am J Public Health. (1993) 83:746–9. doi: 10.2105/AJPH.83.5.746, PMID: 8484463 PMC1694710

[57] Olawale OA Usman JS Oke KI Osundiya OC. Evaluation of predictive factors influencing community reintegration in adult patients with stroke. J Neurosci Rural Pract. (2018) 9:006–10. 10.4103/jnrp.jnrp_386_17 PMC5812161 29456337

[58] O’Donnell AB Travison TG Harris SS Tenover JL McKinlay JB. Testosterone, dehydroepiandrosterone, and physical performance in older men: results from the Massachusetts male aging study. J Clin Endocrinol Metabol. (2006) 91:425–31. doi: 10.1210/jc.2005-1227, PMID: 16332936

